# Unveiling ferroptosis as a promising therapeutic avenue for colorectal cancer and colitis treatment

**DOI:** 10.1016/j.apsb.2024.05.025

**Published:** 2024-05-31

**Authors:** Aaron T. Kao, Christian V. Cabanlong, Kendra Padilla, Xiang Xue

**Affiliations:** Department of Biochemistry and Molecular Biology, University of New Mexico, Albuquerque, NM 87131, USA

**Keywords:** Ferroptosis, Colorectal cancer, Inflammatory bowel disease, Cell death, Molecular mechanisms, Iron metabolism, Drug therapies, Targeted therapeutics

## Abstract

Ferroptosis is a novel type of regulated cell death (RCD) involving iron accumulation and lipid peroxidation. Since its discovery in 2012, various studies have shown that ferroptosis is associated with the pathogenesis of various diseases. Ferroptotic cell death has also been linked to intestinal dysfunction but can act as either a positive or negative regulator of intestinal disease, depending on the cell type and disease context. The continued investigation of mechanisms underlying ferroptosis provides a wealth of potential for developing novel treatments. Considering the growing prevalence of intestinal diseases, particularly colorectal cancer (CRC) and inflammatory bowel disease (IBD), this review article focuses on potential therapeutics targeting the ferroptotic pathway in relation to CRC and IBD.

## Graphical abstract

This review summarizes ferroptosis research progress in colorectal cancer and inflammatory bowel disease, detailing inducers and inhibitors. Ferroptosis emerges as a new strategy for treating enteropathies given the gut's vital role.

**Figure:**
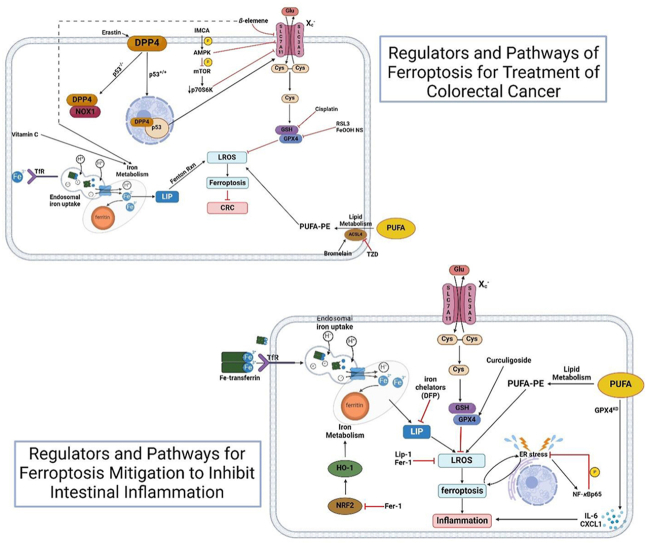

## 1 Introduction

### 1.1 Iron metabolism

**Figure 1:**
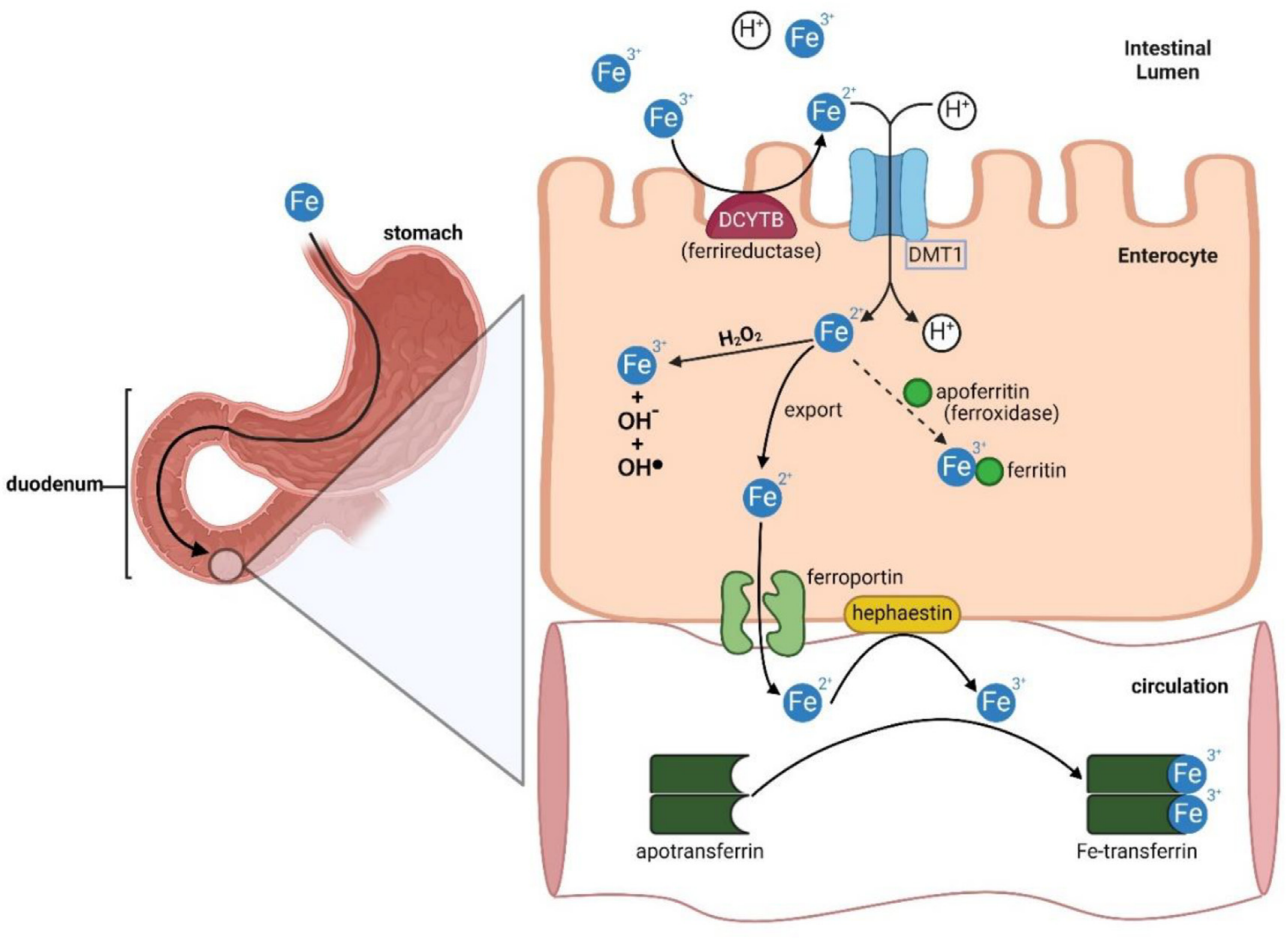
Schematic representation of iron metabolism. Iron absorption is regulated at the apical surface of duodenal enterocytes. Duodenal cytochrome B is a ferrireductase enzyme that catalyzes the reduction of Fe^3+^ iron to Fe^2+^ iron^2^. Divalent metal transporter 1 is a symporter that imports Fe^2+^ and H^+^ in a 1:1 ratio from the intestinal lumen into the enterocyte. Intracellular Fe^2+^ iron reacts with hydrogen peroxide forming reactive oxygen species *via* the Fenton reaction^3^. To prevent iron toxicity, Fe^2+^ iron is oxidized by ferritin heavy chain 1 into inert Fe^3+^ within the ferritin cage^4^^,^^5^. Iron is stored and sequestered within ferritin until released for utilization *via* ferritinophagy^6^. Unstored Fe^2+^ iron is exported out of the enterocyte *via* ferroportin at the basolateral surface of the enterocyte and requires oxidation by hephaestin^7^. In the circulation, the glycoprotein transferrin is capable of binding two Fe^3+^ iron ions for transport to other tissues, utilization in erythropoiesis, or storage in the liver^7^.

Iron is a crucial micronutrient with distinctive chemical properties that play a vital role in various metabolic processes, such as oxygen transport, DNA synthesis, and electron transport. With the ability to exist in both ferric (Fe^3+^) and ferrous (Fe^2+^) states^1^, iron serves a redox function in proteins, facilitating electron transfer. Iron metabolism, illustrated in Fig. 1^2^, ^3^, ^4^, ^5^, ^6^, ^7^, begins with intestinal absorption, and is regulated at the apical surface of duodenal enterocytes. Duodenal cytochrome B (DCYTB), a ferrireductase enzyme, catalyzes the reduction of Fe^3+^ iron to Fe^2+^ iron^2^. Simultaneously, divalent metal transporter 1 (DMT1) imports Fe^2+^ and H^+^ in a 1:1 ratio from the intestinal lumen into enterocytes^3^. Within the cytosol, iron participates in Fenton and Haber-Weiss chemistry, generating potentially harmful reactive oxygen species (ROS) like hydroxyl radicals (OH·)^1^. Iron also catalyzes the formation of organic ROS like lipid peroxides^8^, a characteristic of ferroptosis. To mitigate ROS formation iron is stored in ferritin, an intracellular iron-storage protein containing Ferritin Heavy Chain 1 (FTH1) that has intrinsic ferroxidase activity^4^^,^^5^. Ferritin maintains equilibrium between ferritin-bound iron and the cytosolic transit pool of chelatable iron known as the ‘labile iron pool’ (LIP)^9^. The iron concentration in the LIP is regulated by the cooperative iron efflux activity of ferroportin and hephaestin^10^^,^^11^. Ferroportin (found in duodenal enterocytes and reticuloendothelial cells) facilitates iron efflux. Oppositely, hephaestin (a multi-copper oxidase) oxidizes Fe^2+^ to Fe^3+^, allowing the ferric iron to be stored in transferrin (a glycoprotein capable of binding two Fe^3+^ iron ions until needed for utilization in tissues)^7^^,^^12^. Many of the aforementioned proteins play a crucial role in sequestering and transporting extracellular iron throughout the body and are involved in the process known as ferroptosis.

### 1.2 Ferroptosis

Ferroptosis was first described in 2012 by Dixon et al.^6^, but was initially observed in 2003 by Stockwell et al^13^. In the study Stockwell realized that the drug Erastin (Table 1) caused tumor cells to die in a manner different from apoptosis. Subsequently in 2008, Yang et al. discovered two compounds, RAS-selective lethal (RSL) 3 and RSL5 (Table 1)^13^. Both worked similarly to erastin, and inhibited the drug-induced cell death with the iron chelator deferoxamine (DFO, Table 1), and the antioxidant vitamin E (Table 1)^13^^,^^14^. This confirmed that the new form of cell death is related to the accumulation of intracellular iron and ROS. Morphologically, ferroptosis is distinct from other forms of cell death and is characterized by condensed mitochondria, reduced cristae, and rupturing of the mitochondrial membrane^15^. Ferroptosis can be triggered by the oxidation of phospholipids (lipid peroxidation) found in cellular membranes^14^. The accumulation of lipid peroxides can occur by several mechanisms which include intracellular iron accumulation leading to generation of ROS, inactivation of glutathione peroxidase 4 (GPX4), and inhibition of cystine metabolism and glutathione (GSH) synthesis^14^, ^15^, ^16^, ^17^.

**Table 1:** Structures of chemical compounds important in ferroptosis discovery.

Compound	Structure
Erastin	
RSL3	
Vitamin E	
Deferoxamine	

### 1.3 Colorectal cancer (CRC)

CRC is the third most common malignancy and the second leading cause of death with an estimated 1.9 million incidence cases and 0.9 million deaths worldwide in 2020^18^. The global number of new CRC cases is predicted to reach 3.2 million in 2040, which is attributed to increased population exposure to environmental hazards and the growing prevalence of the Western diet^19^. In the US, despite large-scale screening efforts recommended for all adults, significant numbers of patients are still diagnosed with advanced, metastatic disease due to racial disparities and varying degrees of socioeconomic determinants of health^20^. Treatment for metastatic disease is typically a combination of cytotoxic therapy in conjunction with a targeted therapy, and in spite of recent pharmacologic advancements, the 5-year survival rate is still 12.5%^20^, largely as a result of acquired resistance to therapy occurring in 90% of patients with metastatic cancer^21^. Recent studies have shown that CRC patients with RAS mutations are resistant to biologics, such as monoclonal antibodies to epidermal growth factor receptor (EGFR) or vascular endothelial growth factor, when used in combination with chemotherapy^22^. Additionally, some patients exhibit resistance or intolerance to therapeutics due to apoptosis evasion or anti-apoptotic enhancement^23^^,^^24^, highlighting the importance of developing novel treatments independent of apoptosis as a promising strategy for CRC therapy.

### 1.4 Ferroptosis and colorectal cancer (CRC)

**Figure 2:**
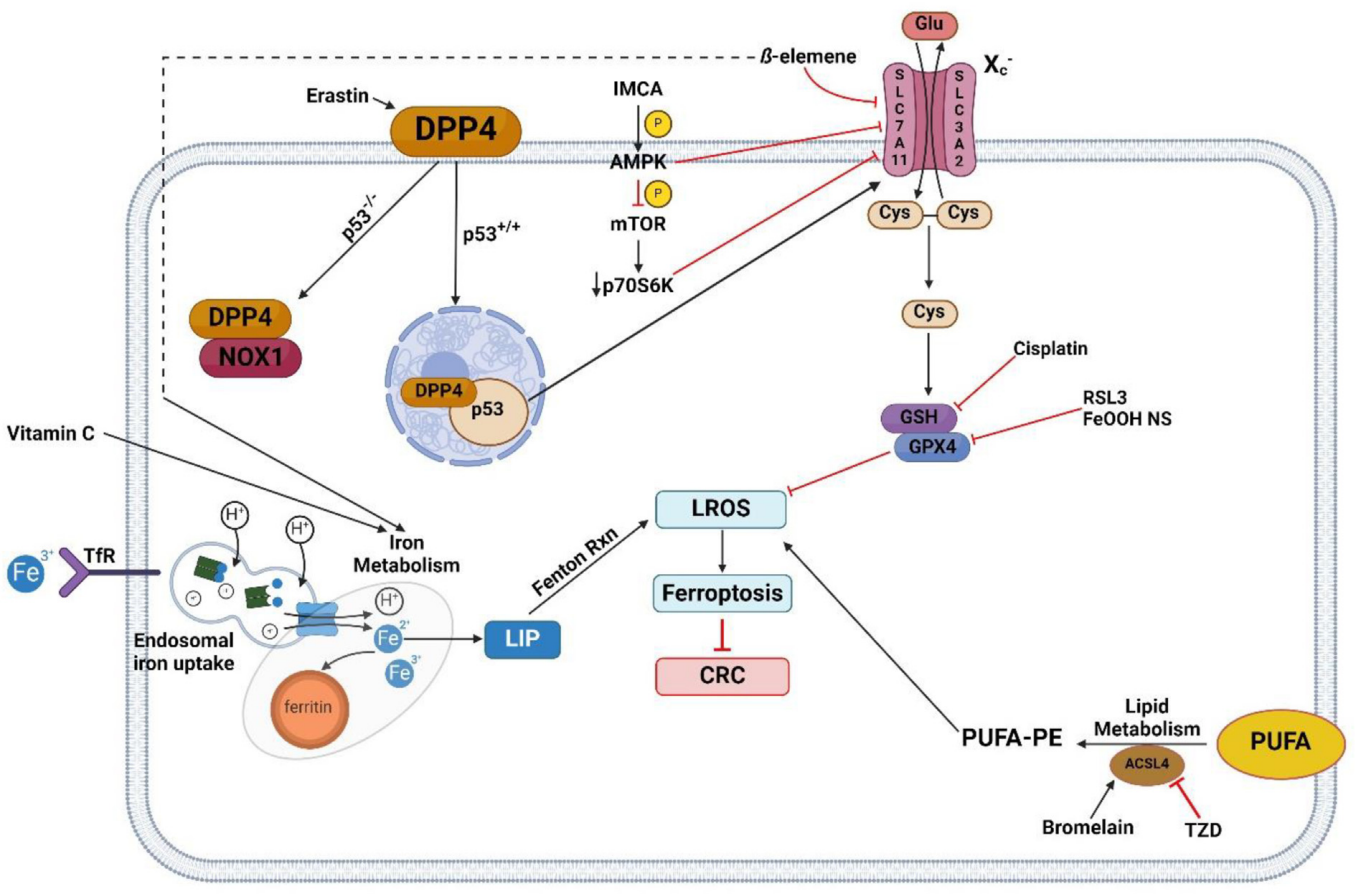
Regulators and pathways of ferroptosis for treatment of colorectal cancer. System X_c_^–^ imports cystine to produce glutathione (GSH)^28^. GSH is used by GPX4 to catalyze the reduction of organic hydroperoxides to form corresponding alcohols, preventing LROS formation and ferroptosis progression^29^. In the context of colorectal cancer treatment, the induction of ferroptosis is desired to kill malignant cells and is accomplished by various compounds that inhibit the GSH–GPX4 pathway, disrupt iron homeostasis, or upregulate ACSL4. In CRC cells, *TP53* prevents ferroptosis by inducing SCL7A11 activity and formation of a p53–DDP4 complex in the nucleus, preventing formation of a DDP4–NOX1 complex which promotes lipid peroxidation^30^^,^^31^. Abbreviations: ACSL4 acyl-CoA, synthetase long chain family member 4; AMPK, AMP-activated protein kinase; CRC, colorectal cancer; Cys, cysteine; DPP4, dipeptidyl-peptidase-4; FeOOH, NS iron oxide hydroxide nanospindle; Glu, glutamate; IMCA, 2-imino-6-methoxy-2*H*-chromene-3-carbothioamide; LIP, labile iron pool; LROS, lipid reactive oxygen species; mTOR, mammalian target of rapamycin; NOX1, NADPH oxidase 1; PUFA, polyunsaturated fatty acids; PUFA-PE, polyunsaturated fatty acid-phosphatidylethanolamine; RSL3, Ras-Selective Lethal 3; SLC3A2, solute carrier family 3 member 2; SLC7A11, solute carrier family 7 member 11; TfR, transferrin receptor; TZD, thiazolidinediones.

The discovery of ferroptosis highlighted its potential as a target for cancer treatment by providing an alternative mechanism to eliminate malignant cancer cells. To date, various tumor cell types including pancreatic ductal adenocarcinoma cells, hepatocellular carcinoma cells, and renal cell carcinoma cells, have been shown to be susceptible to ferroptosis^16^^,^^25^^,^^26^. To induce ferroptosis in cancer cells, two essential events, intracellular iron accumulation and lipid peroxidation, must occur by either inhibition of system X_c_^–^ or inhibition of GPX4^27^ (Table 2, Fig. 2^28^, ^29^, ^30^, ^31^).

**Table 2:** Classification of common ferroptosis modulating chemical compounds.

Classification	Mechanism	Chemical
Inducers	Class 1: inhibit system $X_{c}^{-}$, prevent cysteine import	Erastin, sorafenib, sulfasalazine
	Class 2: inhibit glutathione peroxidase 4	Ras-selective lethal 3 & 5
Inhibitors	Class 1: inhibit accumulation of iron	Deferoxamine, deferiprone
	Class 2: inhibit accumulation of lipid peroxides	Vitamin E, ferrostatin-1, liproxstatin-1

### 1.5 Inflammatory bowel disease (IBD)

IBD is an umbrella term used to describe non-infectious, chronic inflammation of the gastrointestinal tract. It primarily includes ulcerative colitis (UC), which affects only the colonic and rectal mucosa, and Crohn's disease (CD), which can affect any portion of the gastrointestinal tract from the mouth to the anus with transmural inflammation^32^. Although the etiology of IBD is not completely understood, a combination of bacterial contamination, disruption of gut microbiota, uncontrolled immune response, genetic predisposition, and harmful environmental factors has been shown to be associated with the pathogenesis of IBD^33^. IBD commonly presents with symptoms such as abdominal pain, diarrhea, rectal bleeding (more common in UC), weight loss, and perianal disease (the latter two are more common in CD). The typical age of onset is in the second and fourth decades of life, followed by a chronic relapsing pattern^34^. The prevalence of IBD is rising with a retrospective study estimating an increase of incidence from 3.7 million to over 6.8 million cases between 1990 to 2017^35^. Due to the chronic and debilitating nature of IBD in addition to a trend of growing prevalence, the search for novel therapeutic strategies has recently focused on inhibiting ferroptosis to prevent the destruction of intestinal epithelial cells (IEC) and preserve the intestinal mucosal barrier^36^.

### 1.6 Ferroptosis and IBD

Excessive IEC death has been established as a defining characteristic of the pathogenesis of IBD^37^. Iron metabolism dysregulation is also a common feature, and juxtaposed with excessive IEC death, the relationship between ferroptosis and IBD is a topic of recent investigation. A systematic review of IBD revealed that between 8.8% and 73.7% of patients, depending on the subpopulation, develop anemia as a complication of the disease, primarily attributable to iron deficiency^38^. Given the common, generic approach of iron supplementation for iron deficiency, a study examining iron supplementation for CD patients found that oral supplementation may exacerbate intestinal inflammation, correlating this observation with high levels of the pro-inflammatory cytokine IL-6^39^. Similarly, Werner et al.^40^ concluded that supplementation of dietary (luminal) iron was associated with increased IEC stress, inflammation, and cell death in murine models with CD. However, subjects given iron repletion *via* systemic iron supplementation were able to prevent chronic ileitis^40^. Studies on the pathogenesis of UC also found a similar relationship between increased iron consumption and disease progression. Kobayashi et al.^41^ found that high iron intake was correlated to the development in UC in newly diagnosed patients. Additionally, a study by Minaiyan et al.^42^ found that treatment with iron chelator, maltol, was protective against colonic inflammation in colitis-induced rats, indicating iron chelation led to diminished ROS production. These findings suggest a possible role of ferroptosis in IBD pathogenesis and treatment of disease by inhibiting it.

The discovery of an alternative cell death mechanism opened a new avenue for therapeutic discovery for diseases such as CRC and IBD that currently have no available therapeutic treatments. This review proposes possible targets, physiological processes, and compounds that directly or indirectly effect ferroptosis that may be beneficial for gastric diseases.

### 1.7 Classification of ferroptosis modulating chemical compounds (Table 2)

System X_c_^–^ is an amino acid antiporter that imports cystine and exports glutamate in a 1:1 ratio (Fig. 1), and consists of SLC3A2 and SLC7A11 subunits (Fig. 2)^28^. Drugs inhibiting system X_c_^–^, known as Class 1 (Table 1) ferroptosis inducers, disable the import of cystine leading to GSH depletion, inactivation of glutathione peroxidases, and results in reactive oxygen species accumulation (Fig. 2)^16^. In addition to erastin, the U.S. Food and Drug Administration (FDA) approved drugs sorafenib and sulfasalazine belong to this class. Drugs directly inhibiting glutathione peroxidase 4 without glutathione depletion are known as Class 2 ferroptosis inducers, including Ras Selective Lethal 3 and Ras-Selective Lethal 5 (Table 2)^16^. The broadening of ferroptosis research also led to the discovery of inhibitory compounds, which can be separated mechanistically into two categories: those inhibiting iron accumulation (Class I inhibitors including deferoxamine and deferiprone) and those inhibiting lipid peroxidation (Class II inhibitors including vitamin E, ferrostatin-1, liproxstatin-1)^43^.

## 2 Chemical inducers of ferroptosis and CRC therapy

### 2.1 RSL3 and RSL5 (Ras-selective lethal 3, Ras-selective lethal 5, Table 2)

Following the discovery of Erastin, RSL3 and RSL5 were among the first small molecules found to induce ferroptosis (Fig. 2) in cells with oncogenic RAS by binding to and inhibiting GPX4^14^. Given the extensive range of oncogenic RAS signaling, Yang and Stockwell employed the strategy of selective lethal screening by testing 47,725 compounds on various human fibroblast cell lines. The goal was to induce lethality to the cells when coupled with oncogenic RAS^13^^,^^44^^,^^45^. Both RSL3 and RSL5 were positively screened and found to increase lethality with oncogenic RAS in a mechanism that is non-apoptotic, MEK-dependent, iron-dependent, and oxidative with a difference of RSL5 acting in a voltage-dependent anion channel (VDAC)-dependent manner while RSL3 is VDAC-independent^13^. In the context of CRC treatment, a 2018 study tested RSL3 *in vitro* on HCT116, LoVo, and HT29 CRC cells over a 24-h time course. The study found that the molecule initiates ferroptosis in all three cell lines by directly binding and inhibiting GPX4 while increasing transferrin expression^46^. Furthermore, overexpression of *GPX4* rescued RSL3-induced CRC cell death, suggesting a central role for GPX4 in ferroptotic cell death while also confirming RSL3 and RSL5 as possible therapeutic compounds for ferroptosis related diseases^46^.

### 2.2 β-Elemene (Table 3)

While mutations in *RAS* genes represent approximately 25% of all human malignancies, KRAS and NRAS mutations account for approximately 44.7% and 7.5% of CRC cases respectively^47^. Downstream *KRAS* mutations are particularly known to develop drug resistance, leading to the recommendation that anti-EGFR antibodies (*i.e*., cetuximab) in conjunction with chemotherapy be used only for metastatic CRC patients with RAS-wild type^48^. However, a recent study found that *β*-elemene, a bioactive compound isolated from the Chinese herb *Curcumae Rhizoma* possessing anti-proliferative properties, can induce ferroptosis in CRC cells (Fig. 2)^49^^,^^50^. Chen et al. tested the observation that therapy-resistant cancer cells undergoing epithelial-mesenchymal transformation (EMT) were more likely to die *via* induced ferroptosis when compared to nonresistant cancer cells and demonstrated that *β*-elemene in combination cetuximab induced ferroptotic cell death in *KRAS* mutant CRC cells (HCT116 and Lovo)^23^^,^^50^. *KRAS* mutant CRC samples treated with *β*-elemene-cetuximab resulted in low expression of *GPX4*, *SLC7A11*, and ferroportin and high expression of transferrin^23^. Moreover, it was shown that *β*-elemene with cetuximab resulted in downregulation of the mesenchymal markers (Vimentin, N-cadherin, Slug, Snail, and MMP-9) and upregulation of epithelial marker E-cadherin in HCT116 and Lovo cells, indicating the combo treatment may suppress cell migration of *KRAS* mutant CRC cells by inhibiting EMT^23^.

### 2.3 IMCA (2-imino-6-methoxy-2H-chromene-3-carbothioamide, Table 3)

Benzopyran (chromene) is a polycyclic organic that is being considered to treat various diseases and medical applications including diabetes, cancer, and fluorescent probing^51^^,^^52^. IMCA (Fig. 2), a benzopyran derivative with wide ranging biological activity, has been studied for therapeutic use in cancer, type 2 diabetes, inflammation, skin diseases, Alzheimer's disease, polycystic kidney disease, and viral/bacterial infections^53^. In 2019, Zhang et al.^54^ demonstrated that IMCA exposure induced the death of CRC cells *in vitro* and inhibited tumor growth in murine models with negligible organ toxicity. Their study found that IMCA regulated the activity of the AMP-activated protein kinase (AMPK)/mammalian target of rapamycin (mTOR)/p70S6K signaling pathway resulting in inhibition of the SLC7A11 subunit of system X_c_^–^ leading to ferroptosis, whereas overexpression of *SLC7A11* resulted in rescue of IMCA-induced ferroptosis^54^. IMCA phosphorylates and activates AMPK, which is a negative regulator of mTOR; *p*-AMPK then phosphorylates and inhibits mTOR, resulting in decreased activity of the downstream target protein p70S6K, a critical enzyme involved in protein synthesis, cell proliferation, and autophagy^54^^,^^55^. The known morbidity and mortality associated with chemotherapy for CRC following surgery necessitates alternative therapeutics with high efficacy and low toxicity, and IMCA may be a likely candidate for this criterion.

### 2.4 Cisplatin (Table 3, Fig. 3)

**Figure 3:**
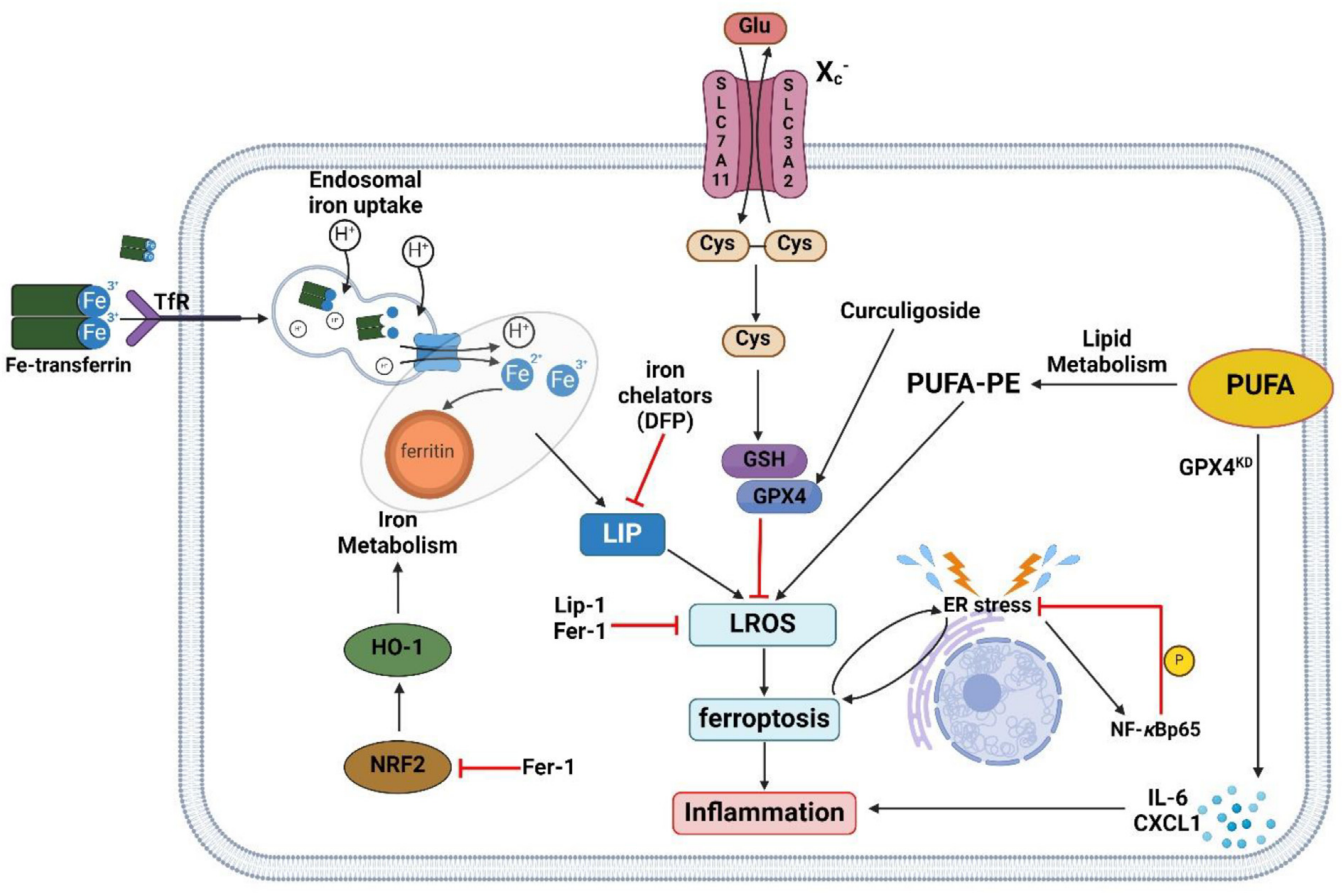
Regulators and pathways for ferroptosis mitigation to inhibit intestinal inflammation. System X_c_^–^ imports cystine to produce GSH^28^. GSH is used by GPX4, in the cytoplasm, to catalyze the reduction of organic hydroperoxides to corresponding alcohols, preventing formation of LROS, ferroptosis progression, and mitigating inflammation^29^. Iron chelators bind free iron, limiting the LIP and generation of ROS by Fenton reaction. Ferrostatin-1 and liproxstatin-1 are synthetic radical trapping antioxidants and known ferroptosis inhibits^68^. Deferiprone is an oral iron chelator and binds free iron to reduce the LIP and decreases ROS production. Ferrostatin-1 administration decreases Nrf2 expression, which leads to downregulation of HO-1 and decreases iron turnover and accumulation^68^. Mice with reduced GPX4 expression fed a Western diet rich PUFAs/*ω*-6 fatty acids preferentially oxidized arachidonic acid, generating high levels of inflammatory mediators IL-6 and CXCL1^69^. ER stress and ferroptosis feedback, exacerbating each other. ER stress also activates NF-*κ*B, and phosphorylation of the NF-*κ*B p65 subunit promotes negative feedback on ER stress^70^. Abbreviations: Cys, cysteine; CXCL1, C–X–C motif ligand 1; DFP, deferiprone; ER, endoplasmic reticulum; Fer-1, ferrostatin-1; Glu, glutamate; GSH, glutathione; GPX4, glutathione peroxidase 4; GPX4^KD^, glutathione peroxidase 4 knockout; HO-1, heme-oxygenase 1; IL-6, interleukin-6; LIP, labile iron pool; Lip-1, liproxstatin-1; LROS, lipid reactive oxygen species; NF-*κ*Bp65, Nuclear factor-*κ*B; NRF2, nuclear factor erythroid 2-related factor 2; PUFA, polyunsaturated fatty acids; PUFA-PE, polyunsaturated fatty acid-phosphatidylethanolamine; SLC3A2, solute carrier family 3 member 2; SLC7A11, solute carrier family 7 member 11; TfR, transferrin receptor.

In a 2018 study conducted by Guo and colleagues^24^, classic chemotherapeutic drugs were screened for their ability to induce ferroptosis (Fig. 3), and cisplatin was notable as an inducer of both ferroptosis and apoptosis in HCT116 cells. The study verified the presence of ferroptosis by demonstrating that cisplatin-induced cell death was partially reversed by addition of ferrostatin-1, DFO, and *β*-mercaptoethanol. Further confirmation of ferroptosis occurrence was conducted by observing mitochondrial structural changes, partial cytotoxic reversal of cisplatin by transfection of iron-responsive binding element 2 (IREB2) siRNA, and measurement of ROS levels *via* flow cytometry analysis. The study also found that combination therapy of cisplatin and erastin had a synergistic anti-tumor effect on both HCT116 and A549 cells.

### 2.5 Vitamin C (Table 3)

Much like other vitamins, vitamin C is multifaceted having roles in various physiological pathways. In particular, vitamin C plays a role in collagen synthesis, hormone synthesis, regulation of transcription, translation, protection against reactive oxygen species, and reduction of gastrointestinal iron^56^. Vitamin C (ascorbate) facilitates the absorption of nonheme iron (Fig. 2) in the small intestine and is a modulator of iron metabolism by stimulating ferritin synthesis, inhibiting lysosomal ferritin degradation, and decreasing cellular iron reflux under normal physiological conditions^57^. While the antioxidant function of ascorbate is widely known, new studies discuss a paradoxical characteristic of ascorbate due to its toxicity to cancer cells and pro-oxidative activity by inducing H_2_O_2_ production and oxidative stress^58^. Studies have described a mechanism of ascorbate and superoxide-mediated ferritin iron reduction and intracellular iron release^59^^,^^60^. The pro-oxidative activity of ascorbate is iron-dependent and activated in cancer cells^58^. Within cancer cells, iron levels are elevated to facilitate adaptation to hypoxia and stimulate cell proliferation, whereas in normal cells, the antioxidative function of ascorbate dominates due to a limited LIP^61^. Research published by Chen et al.^62^ demonstrated pharmacologic intravenous dosing of ascorbate selectively kills cancer cells (but not normal cells) *via* extracellular generation of H_2_O_2_. These findings are encouraging for drug-resistant CRC, which is resistant to anti-EGFR biologics such as cetuximab due to ‘persister’ cells, but new evidence suggests combo therapy of cetuximab with pharmacologic dosing of ascorbate impairs the emergence of persister cells, limits growth of CRC organoids, and significantly delays acquired resistance by triggering adenosine triphosphate depletion and oxidative stress resulting in ferroptotic cell death^63^. Moreover, the study by Lorenzato and colleagues^63^ found that ascorbate–cetuximab combo in CRC cell lines triggered ferroptosis by increasing ROS production, sustaining ROS persistence, increasing ferritin levels, increasing reduction of ferritin and iron release, and decreasing GSH levels. Intravenous pharmacologic dosing of ascorbate adds little to no toxicity to an already approved cetuximab regimen, although more research is needed to establish optimal dosing and scheduling for clinical use.

### 2.6 Iron oxide-hydroxide nanospindles

A novel, multifunctional approach to CRC treatment was proposed by Li et al.^64^ involving engineered nanomaterials to scavenge endogenous hydrogen sulfide (H_2_S) *via* reductive chemistry to prohibit cancer growth. Endogenous H_2_S production has been shown in various studies to be the result of cystathione-*β*-synthase overexpression in malignant colon and ovarian cells leading to stimulation of proliferative, migratory, and invasive signaling pathways in addition to enhanced tumor angiogenesis^65^. The proposed iron oxide-hydroxide nanospindle (FeOOH NS) serves multiple functions: (1) as a paramagnetic agent, FeOOH NSs have been utilized as a magnetic resonance imaging contrast agent for cancer diagnosis^66^. (2) FeOOH NSs are efficient scavengers of H_2_S gas with high reactivity and adsorption capacity of H_2_S under room temperature and ambient pressure^67^. (3) FeOOH NS treatment with near-infrared photothermal therapy resulted in production of iron sulfide (FeS), which was shown to selectively kill CT26 colon cancer cells with near-infrared laser irradiation and induced ferroptosis by inhibiting GPX4^64^.

## 3 Chemical inhibitors of ferroptosis and IBD therapy

### 3.1 Ferrostatin-1, liproxstatin-1, and DFP

A 2020 study by Chen et al.^68^ examined the role of ferroptosis in the pathogenesis of UC. Researchers used a commonly employed method to study UC by inducing colitis in murine models by administration of dextran sodium sulfate (DSS) in drinking water, which induces intestinal inflammation from an unclear mechanism, but most closely resembles human UC. The authors observed that mice with acute DSS-induced colitis displayed upregulation of cyclooxygenase-2 (COX-2), resulting in a pro-inflammatory state due to increased prostaglandins. Additionally, there was an upregulation of acyl-CoA synthase long-chain family member 4 (ACSL4), indicating greater ferroptotic sensitivity^68^. Histopathological analysis found increased epithelial inflammatory infiltration and necrosis with distinct distortion of intestinal crypts. Subsequent administration of ferrostatin-1 and liproxstatin-1 (Table 4), synthetic radical trapping antioxidants (RTAs) and inhibitors of lipid peroxidation/ferroptosis (Fig. 3), resulted in a decreased inflammation index, alleviation of microscopic colon damage, and reduced MDA levels compared to 3% DSS treated mice^68^. Moreover, the administration of DFP (Table 4), an orally active iron-chelating agent, also resulted in decreased MDA and intestinal inflammation compared to only 3% DSS-treated mice, suggesting a reduction in iron levels positively affects DSS-induced UC^68^. These results demonstrate the role of ferroptosis in colitis and suggest therapy with ferroptosis inhibitors and iron chelation may be effective treatment (Fig. 3).

**Table 3:** Structures of ferroptosis inducers important in colorectal cancer therapy.

Compound	Structure
*β*-Elemene	
IMCA	
Cisplatin	
Vitamin C	
Sulfasalazine	
Sorafenib

**Table 4:** Structures of ferroptosis inhibitors important in inflammatory bowel disease therapy.

Compound	Structure
Ferrostatin-1	
Liproxstatin-1	
Deferiprone	
Vitamin K hydroquinone

**Table 5:** Common gene modulators of ferroptosis important in colorectal cancer therapy.

Classification	Molecule	Mechanism
Enhancers	*ACSL4*	Uses polyunsaturated fatty acids (PUFAs) as a substrate to create phospholipids.
	*TP53*	Prevents lipid peroxidation at the plasma membrane by sequestering DPP4 it into the nucleus.
Inhibitors	*FSP1*	Reduces ubiquinone (CoQ_10_) to ubiquinol (CoQH_2_) and sequesters reducing factors into the plasma membrane to prevent the accumulation of lipid peroxides.
	*DHODH*	Reduces ubiquinone (CoQ_10_) to ubiquinol (CoQH_2_) and sequesters reducing factors into the mitochondria to prevent the accumulation of lipid peroxides.

**Table 6:** Common gene inhibitors of ferroptosis important in IBD therapy.

Molecule	Mechanism
*GPX4* (glutathione peroxidase 4)	Converts phospholipid peroxides into hydroxylated phospholipid compounds.
*NRF2* (nuclear factor erythroid 2-related factor 2)	Transcribes antioxidant proteins and enzymes in response to oxidative stress.
*NF-κBP65* (nuclear factor erythroid 2-related factor 2)	One of the five subunits of NF-*κ*B involved in ER stress mediated apoptosis.

### 3.2 Vitamin K (Vk, Fig. 4)

**Figure 4:**
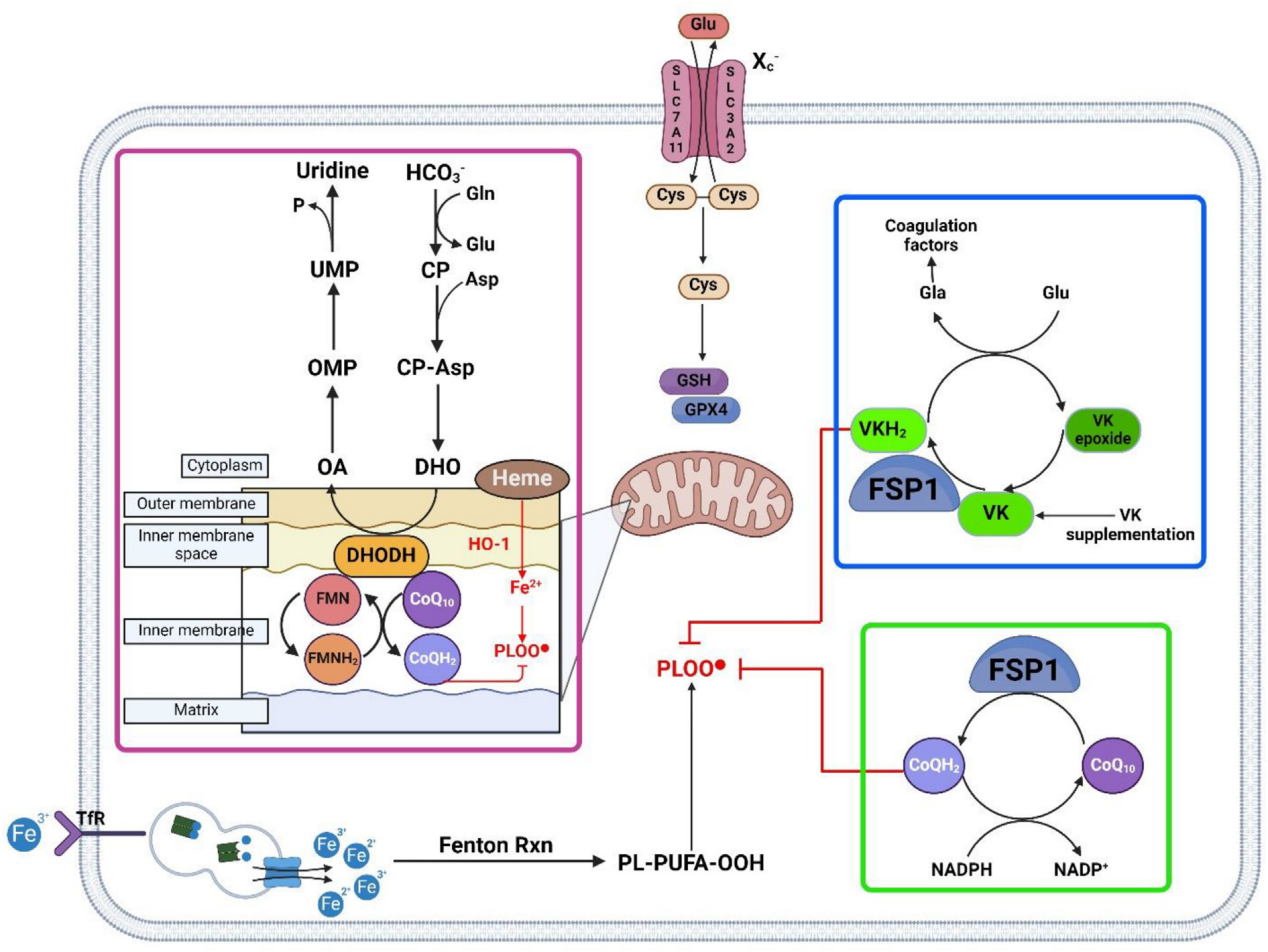
Non-canonical GPX4 independent ferroptosis suppressor protein 1 (FSP1) and dihydroorotate dehydrogenase (DHODH) anti-ferroptosis pathways important in CRC therapy. The green border demarcates FSP1 possessing NADH:ubiquinone oxidoreductase activity, which reduces ubiquinone (CoQ10) to form ubiquinol (CoQH2) using nicotinamide adenine dinucleotide phosphate (NADPH)^78^. CoQH2 is a lipophilic radical-trapping antioxidant (RTA) and prevents lipid peroxidation^79^. In the blue, FPS1 also acts as a vitamin K reductase and generates reduced vitamin K hydroquinone (VKH_2_), which functions as an RTA^72^. The pink border demarcates DHODH, known for its role in *de novo* pyrimidine synthesis, which is embedded in the mitochondrial inner membrane and generates CoQH2 to detoxify lipid peroxides^80^. Abbreviations: Asp, aspartate; CoQ10, ubiquinone; CoQH2, ubiquinol; CP, carbamoyl phosphate; CP-Asp, carbamoyl aspartic acid; Cys, cysteine; DHO, dihydroorotate; DHODH, dihydroorotate dehydrogenase; FMN, flavin mononucleotide; FMNH_2_, reduced flavin mononucleotide; FSP1, ferroptosis suppressor protein 1; Gla, *γ*-carboxyglutamate; Glu, glutamate; GSH, glutathione; GPX4, glutathione peroxidase 4; HCO_3_^–^, bicarbonate; HO-1, heme-oxygenase 1; OA, orotate; OMP, orotidine-monophosphate; P, phosphate; PLOO^·^, phospholipid hydroperoxyl radical; PL-PUFA-OOH, phospholipid-polyunsaturated fatty acid-hydroperoxide; SLC3A2, solute carrier family 3 member 2; SLC7A11, solute carrier family 7 member 11; TfR, transferrin receptor; UMP, uridine-monophosphate; VitK, vitamin K; VitKH_2_, vitamin K hydroquinone.

The vitamin K is a known cofactor for *γ*-glutamyl carboxylase and is vital to the coagulation process as many of the proteins involved are dependent on vitamin K to function (Fig. 4)^71^. The search for additional cellular-intrinsic factors that determine ferroptosis sensitivity led to the discovery of anti-ferroptotic properties of reduced Vk, Vk hydroquinone (VKH_2_) (Table 4). In a study screening for naturally available vitamins that also prevent ferroptosis, Mishima et al.^72^ discovered three variants of reduced Vk [phylloquinone, menaquinone-4 (MK4), and menadione] in addition to *α*-tocopherol (the most bioactive form of vitamin E) that rescued cells from tamoxifen-induced deletion of GPX4 in Pfa1 cells. Additionally, the Vk variants were also shown to prevent ferroptosis in human cancer A375 and 786-O cell lines lacking *GPX4* expression and rescued RSL3-induced GPX4 inhibition in fibrosarcoma HT-1080 cells^72^. MK4, produced by gut bacteria and derived from phylloquinone in plants^73^, was notably found to prevent ferroptosis *in vivo* from hepatocytes of mice with tamoxifen-induced GPX4 deletion^72^. Mishima et al. also uncovered an alternate pathway of Vk's reduction to VKH_2_ that is resistant to warfarin, an anticoagulant that inhibits Vk epoxide reductase. Due to the structural similarities of ubiquinone and Vk, as well as both molecules' ability to function as an RTA, Mishima et al. tested and confirmed the ability of FSP1 to act as a Vk reductase (Fig. 4). This finding presents an FSP1-dependent, non-canonical pathway of the vitamin K cycle that can protect against lipid peroxidation and ferroptosis^72^. Based on their findings, Mishima and co-authors suggest that menaquinone may have been the first naturally occurring anti-ferroptotic quinone. This was later substituted by CoQ10 following the Great Oxidation Event of the Paleoproterozoic era due to CoQ10's greater redox potential and increased abundance (Fig. 4)^74^^,^^75^.

Vk and its influence on the ferroptosis pathway are very interesting, but the relevance toward CRC therapy is made more evident by the variants of Vk currently tested on CRC cells. One example of this is the Vk variant called Manadione. This catabolic product of Vk has shown to be toxic to human cancer cell lines (SW480 and SW620), but non-toxic to primary colon epithelial cells^76^. Additionally, manadione was shown to suppress EMT transition, inhibit Wnt signaling pathways, and inhibit differentiation in CRC cells^76^. Another example shows that bacterial produced VK can induce apoptosis in a human (Caco-2) CRC cell line. The study described in Kishore et al. tested the bacterial Vk and showed that they could induce around 13% reduced viability in CRC cultures at around 20 μmol/L^76^. A similar study by Orlando et al.^77^ took the idea further and co-treated with Vk and Vk producing bacteria. The results show that this combination had proapoptotic and anti-proliferative effects in all three CRC cell lines: Caco-2, HT-29, and SW480. Interestingly, when administered separately Vk was only able to have these effects in two of the three CRC cell line suggesting some additive or synergistic effects of the bacteria^56^. Ultimately the collective studies show that Vk and its many variants directly affect CRC cells negatively, affirming Vk as a novel therapeutic with potential.

## 4 Genetic modulators of ferroptosis and CRC therapy

### 4.1 ACSL4 (Table 5)

Long-chain acyl-CoA synthetases are known to catalyze the thioesterification of long-chain fatty acids into their respective acyl-CoA derivates. A 2017 study by Doll et al.^81^ demonstrated the isoform acyl-CoA synthase long-chain family member 4 (ACSL4) was essential for ferroptosis execution. ACSL4 is a regulator of lipid production in intact cells, ligating long-chain polyunsaturated fatty acids (PUFAs) (Fig. 2), which is preferential to arachidonic acid (AA), an *ω*-6 fatty acid (FA), as a substrate for oxidation^82^^,^^83^. Doll et al.^81^ confirmed *Acsl4*, as an essential pro-ferroptotic gene by showing *GPX4/ACSL4* double knockout cells, were viable for a period over 10 days. Also, studies have found that thiazolidinediones, used to increase insulin sensitivity in type 2 diabetes, have a therapeutic effect by inhibiting ACSL4 aside from its effect on PPAR-*γ* and have been shown to alleviate progression of ferroptosis-related disease including neurodegeneration and ischemia/reperfusion injury^84^^,^^85^. In the context of CRC treatment, a study by Park et al.^86^ found that treatment with bromelain in *KRAS* mutant murine models induced ferroptotic death in CRC cells by induction and upregulation of *ACLS4*. These results suggest *ACSL4* inhibition as a therapeutic approach may prevent ferroptosis in relevant diseases.

### 4.2 TP53 (Table 5, Fig. 2)

Although ferroptosis was initially described occurring in *RAS* mutant cancer cells, recent studies have determined that ferroptosis is not unique to the *RAS* pathway and show an unexpected role for *TP53* in promoting or inhibiting ferroptosis under different cellular conditions^13^^,^^87^. *TP53*, the known ‘guardian of the genome’ and tumor suppressor, participates in autophagy, apoptosis, and the cell cycle, but also regulates ferroptosis by transcriptional or post-transcriptional mechanisms^88^. In non-CRC cells, *TP53* induces ferroptosis by inhibiting transcription of the *SLC7A11* gene, preventing formation of its respective subunit of system X_c_^–^ (Fig. 2), blocking cystine import, and suppressing GPX4 activity resulting in increased ferroptosis sensitivity due to oxidant insults^89^. On the other hand in CRC cells, TP53 stimulates *SLC7A11* expression to inhibit ferroptosis and was also found to have a direct molecular link with dipeptidyl-peptidase 4 (DPP4), a multifunctional transmembrane protein ubiquitously expressed on the surface of a variety of cells^16^^,^^30^. DPP4 is also a serine protease that cleaves a variety of substrates, notably incretin hormones, and is implicated in a variety of diseases^90^. Research by Xie et al.^84^ demonstrated that TP53 inhibits erastin-induced ferroptotic CRC cell death by formation of a P53–DPP4 complex in the nucleus, blocking DPP4 activity, and disassembly of this complex restores the erastin-induced ferroptosis sensitivity^30^^,^^31^. However, the loss of *TP53* prevents DPP4 nuclear localization and facilitates the formation of the DPP4–NADPH oxidase 1 (NOX1) complex that promotes lipid peroxidation, resulting in ferroptosis in CRC cells^30^.

### 4.3 Ferroptosis suppressor protein 1 (FSP1, Table 5, Fig. 4)

While the significance of GPX4 in ferroptosis sensitivity has been established, the ability of *GPX4* inhibition to induce ferroptosis is still largely unknown given the range of cancer cell types with varying degrees of inherent resistance across different organ systems^91^. It has also been shown that inhibition of *GPX4* was ineffective to trigger ferroptosis regardless of *ACLS4* expression in some cancer cell lines, suggesting additional, undiscovered factors involved in ferroptosis resistance^81^^,^^92^. To identify these factors, a study by Doll and colleagues conducted a cDNA screen of ferroptosis-resistant cell lines complementing loss of *GPX4* and identified seven clones expressing apoptosis-inducing factor mitochondria-associated 2 (*AIFM2*), which codes for a flavoprotein originally thought to be an inducer of apoptosis^93^. *AIFM2* overexpression in mouse embryonic Pfa1 cells and human fibrosarcoma HT1080 cells resulted in robust protection against both pharmacologic and genetic inducers of ferroptosis, and given these findings, Doll et al.^94^ recommended future reference of AIFM2 as FSP1. Doll et al. found that FSP1's anti-ferroptotic activity was independent of cellular GSH levels, GPX4 activity, *ACLS4* expression, and oxidizable fatty acid content, conferring protection specific to ferroptosis-inducing agents but no protection against cytotoxic compounds or pro-apoptotic conditions^94^. Furthermore, in agreement with the previous study, Bersuker et al.^95^ confirmed in lung tissue that FSP1 (Fig. 4) suppresses ferroptosis through its NADH:ubiquinone oxidoreductase activity. This activity reduces ubiquinone (CoQ10) to form ubiquinol (CoQH2), which is known as an effective radical-trapping antioxidant (RTA) in phospholipids and lipoproteins^78^^,^^79^^,^^95^. Furthermore, FSP1 (Fig. 4) catalyzes the regeneration of CoQ_10_ from CoQH_2_ using nicotinamide adenine dinucleotide phosphate (NADPH)^94^. The discovery of FSP1 sheds light on an important mechanism whereby cells can evade ferroptosis induction in a manner independent of the canonical ferroptosis pathway and elucidates an important factor in cancer resistance to induced ferroptotic cell death.

*FSP1* has importance as a potential target for CRC therapeutics because there is evidence that it may play a predictive role in determining the long-term outcome of CRC patients. In a study done by Im et al.^96^ examining the role of *FSP1* in a cohort of 135 CRC patients found that cells expressing *FSP1* correlated with a short 10-year survival outcome. Additionally, they found through a network analysis that that *FSP1* expression in these patients significantly correlated to cancer invasiveness factors^96^. Additional evidence for *FSP1* role in invasion comes from Gardillo et al.^97^ where they examined sections from two groups of patients with invasive or superficial intestinal adenocarcinomas. This study showed that FSP1 along with *α*-SMA was used to easily identify cancer-associated fibroblast in patients. Interestingly they also saw that FSP1 was confined to the transitioning fibroblast highlighting FSP1 as a potential biomarker and target in the early stages CRC^97^. The therapeutic capacity of *FSP1* is shown *in vitro* by a study involving curcumin by Miyazaki et al.^98^, the paper shows curcumin treatment induces ferroptosis by suppressing *FSP1* and *GPX4*. In this study curcumin treatment reduced the cell viability of SW480 and HCT116 cell lines showing *FSP1* to be a promising target for CRC therapy^98^.

### 4.4 Dihydroorotate dehydrogenase (DHODH, Table 5, Fig. 4)

Following discovery of FSP1's role in ferroptosis resistance, researchers found a novel function for mitochondrial enzyme DHODH, known for its essential role in *de novo* synthesis of pyrimidines (Fig. 4). The fourth step of *de novo* pyrimidine synthesis is catalyzed by DHODH, requiring ubiquinone-mediated oxidative activity to convert dihydroorotate to orotate, and produces ubiquinol. Research by Mao et al.^80^ reported that DHODH by way of ubiquinol works alongside GPX4 to detoxify lipid peroxide accumulation within mitochondria. The DHODH inhibitor brequinar was found to selectively suppress tumor growth by inducing ferroptosis in tumors with decreased *GPX4* expression. The combination treatment of brequinar with sulfasalazine (Table 3), an FDA-approved drug with ferroptosis-inducing activity, resulted in synergistic induction of ferroptosis in tumors with high *GPX4* expression^80^. Mao's findings^97^ provide a new perspective on how mitochondria prevent ferroptosis *via* a GSH-independent enzyme and suggest DHODH as a potential therapeutic target to treat cancers expressing low *GPX4*^99^.

## 5 Genetic inhibitors of ferroptosis and IBD therapy

### 5.1 GPX4 (Table 6)

Following import into the cell by system X_c_^–^, cystine is immediately reduced to cysteine, the rate-limiting step for GSH synthesis^100^. Cysteine's thiol group gives the molecule specialized properties involved in reduction and conjugation reactions needed for removal of peroxides and various xenobiotic compounds^101^. GPXs are a family of phylogenetically related enzymes that catalyze the reduction of H_2_O_2_ and organic hydroperoxides to water and corresponding alcohols using reduced GSH as an electron donor^29^. Of the GPX family, GPX4 plays a central role in ferroptotic cell death as it appears to be protective against oxidative damage to phospholipids within cellular membranes^102^. GPX4 primarily exists in its cytoplasmic variant but it can also be found in low amounts in the mitochondria (Figure 2, Figure 3, Figure 4). Notably, a 2018 study found an indispensable role for selenium in mammalian embryogenesis by showing selenocysteine-containing GPX4, rather than cysteine-containing GPX4, provided superior resistance to overoxidation and enhanced protection against peroxide-induced ferroptosis^103^, further highlighting GPX4's central role in preventing ferroptosis. In the context of IBD, functional expression of *GPX4* negatively regulates the ferroptotic pathway, which is desirable to reduce intestinal inflammation. Mayr et al.^69^ examined the role of GPX4 in an IBD compared biopsy-derived IECs from non-IBD patients to lesioned and non-lesioned mucosa of CD and UC patients and found that *GPX4* expression was decreased in CD patients but was indistinguishable between healthy and UC patients. It was discovered that mice lacking one *GPX4* allele (*GPX4*^+/−IEC^) exposed to a Western diet rich in PUFAs/*ω*-6 fatty acids developed signs of epithelial lipid peroxidation and focal neutrophilic enteritis with granulomatous accumulation of inflammatory cells, exemplifying how a Western diet high in PUFAs contributes to an increased risk of developing CD^69^. Additionally, IECs with reduced *GPX4* expression resulted in the lipid peroxidation of AA, and furthermore, AA was found to induce the expression of interleukin-6 (*IL-6*) and C–X–C motif ligand 1 (*CXCL1*) while stimulating enzymatic peroxidation *via* lipoxygenase^69^. In congruence with a prior 2016 study by Yang and co-authors, Mayr et al.^69^ concluded that iron availability promoted PUFA-induced lipid peroxidation when *GPX4* expression is reduced, demonstrating that ferroptotic mechanisms drive intestinal inflammation in IECs. Interestingly, Mayr et al.^69^ found that cell death was not necessary for the development of intestinal inflammation, indicating decreased *GPX4* expression as represented by *GPX4*^+/−IEC^ mice may be protective against ferroptotic cell death, but is insufficient to prevent PUFA-induced inflammation and lipid peroxidation. Given that decreased GPX4 activity was found to promote ferroptotic changes in cells modeling CD, a different study on UC found that a specific plant compound was protective against ferroptotic changes by upregulating *GPX4* while enhancing its antioxidant activity. Curculigoside is a naturally occurring compound found in the Curculio orchioides flowering plant and has been utilized in traditional medicines across various cultures for ailments ranging from arthritis and impotence to hypertension and diarrhea^104^. Given its history of use as a natural remedy for gastrointestinal disease, curculigoside was the subject of study by administration to mice with DSS-induced colitis. This study by Wang et al.^105^ found that administration of curculigoside in a 7-day period reversed ferroptotic alterations (Fig. 3) in the IECs of colitis-induced mice by promoting *GPX4* transcription in IEC-6 cells while increasing GPX4 selenium sensitivity.

### 5.2 Nuclear factor erythroid 2-related factor 2 (NRF2, Table 6)

NRF2 is a transcription factor that is activated by ROS or external stimuli (Fig. 3)^98^. NRF2 activation by oxidative stress results in translocation to the nucleus, binding with the antioxidant response element (ARE), and transcription of genes for various protective enzymes, including Phase 2 detoxifying enzymes, among which includes heme-oxygenase 1 (*HO-1*)^106^. Though HO-1 is widely known for its role in heme degradation, it has been found to have a dual role that is cytoprotective effect against various stress-related conditions, and conversely, is a causative factor for the progression of several diseases^107^. A study by Chen et al.^68^ demonstrated that oxidative stress resulting from ferroptosis may upregulate *NRF2* and *HO-1* expression. When the ferroptosis inhibitor ferrostatin-1 was administered to 3% DSS mice, the expression of the *NRF2-HO-1* signaling pathway was decreased. The decreased expression of *NRF2/HO-1* because of ferrostatin-1 administration suggests that ferroptotic progression from DSS-induced colitis activates NRF2-ARE by oxidative stress signaling. This activation leads to the overexpression of *HO-1* and causes iron accumulation^108^. Targeting *NRF2* in UC may attenuate oxidative stress and ferroptotic progression by halting NRF2–HO-1 from generating more free iron.

### 5.3 Nuclear factor-κB (NF-κB, Table 6)

NF-*κ*B is a family of inducible transcription factors that mediate inflammatory responses and is involved in various aspects of innate and adaptive immune functions^109^. Prior studies have shown that NF-*κ*B is protective to IECs by promoting resistance to cell damage and death. Qiu et al.^70^ demonstrated that *in vivo* administration of BAY 117085, an inhibitor of NF-*κ*B, led to IEC apoptosis. Additional studies conducting gene deletion of *NF-κB* members in mice found greater susceptibility of IECs to inflammation and death^110^^,^^111^. Further investigation by Xu et al.^112^ found for the first time that the heterodimer subunit NF-*κ*B p65 conferred a protective role against ferroptosis in mice with DSS-induced colitis through direct interaction with eIF2A. The study found that NF-*κ*B p65 suppressed ferroptosis by reduction of endoplasmic reticulum (ER) stress (Fig. 3), consistent with previous literature, but also found that DSS-treated NF-*κ*B P65^IEC KO^ mice displayed concurrent elevation of phosphorylated-eIF2A (an ER stress signaling protein) and FTH1, providing a new perspective to recognize ER stress-mediated ferroptosis^112^. Furthermore, the study found that administration of ER stress inducers thapsigargin and tunicamycin to HCoEpiC cells led to activation of NF-*κ*B p65 and subsequent increased levels of phosphorylated NF-*κ*B p65, prompting negative feedback on ER stress and protecting against ferroptosis^112^. The study concluded that phosphorylation of NF-*κ*B p65 is a potential therapeutic target for UC.

## 6 Cell physiological processes as an avenue for novel therapeutics

### 6.1 ER stress

The ER stress response is an essential cellular mechanism that attenuates protein synthesis and decreases the burden on the ER when misfolded/unfolded proteins accumulate and exceed degradation capacity^113^. The end result of irreversible ER stress is the induction of apoptosis to dispose of injured cells, and the malfunctioning of this system has been implicated in diabetes, inflammation, and neurodegenerative diseases^114^. Researchers of IBD have known that ER stress is related to inflammation of intestinal mucosa^115^^,^^116^, but more recent studies have shown that ferroptotic agents induce ER stress and lead to activation of pro-apoptotic proteins^117^. The ER stress response is monitored by three upstream signaling proteins: inositol requiring protein-1, activating transcription factor-6, and protein kinase RNA-like ER kinase (PERK), and when activated, these proteins induce apoptosis^118^. Additionally, the ER stress indicator activating transcription factor 4 (ATF4) is known to regulate several ER stress response target genes^119^. The interaction of these proteins represents a cross-point for various signaling pathways. Notably, the phosphorylation of PERK inactivates the *α*-subunit of eukaryotic translational initiation factor 2 (eIF2A) leading to translational attenuation, which interestingly results in the upregulation of *ATF4*^117^. The resultant upregulation of *ATF4* causes increased translation of CCAAT-enhancer-binding protein homologous protein (CHOP), a transcription factor involved in inducing apoptosis^120^. During times of ER stress, CHOP binds to the promoter of pro-apoptotic protein p53 upregulated modulator of apoptosis (PUMA) and increases its expression^121^. The exact mechanism of IEC death due to ER stress remains unclear as Lee et al.^117^ found that the ferroptosis-inducing agent and anti-malarial drug artesunate resulted in greater PUMA expression without inducing apoptosis. However, Park et al.^122^ found that whole cigarette smoke condensates induced ferroptosis in bronchial epithelial cells by triggering ER stress. Additionally, a study by Xu et al.^112^ further tested this mechanism by administration of GSK 414, an inhibitor of PERK, to IECs of mice with induced colitis both *in vivo* and *in vitro* and found a significant decrease in IEC cell death, iron content, and ROS, suggesting that inhibition of ER stress signaling was protective against ferroptotic cell death. Moreover, Xu et al.^112^ found that human colonic epithelial cells (HCoEpiC) pre-treated with GSK 414 and challenged with RSL3 were rescued from ferroptosis. These findings indicate that ER stress contributes to apoptosis signaling in IECs with UC and also stimulates ferroptosis in an unclear mechanism.

### 6.2 Ferritinophagy

**Figure 5:**
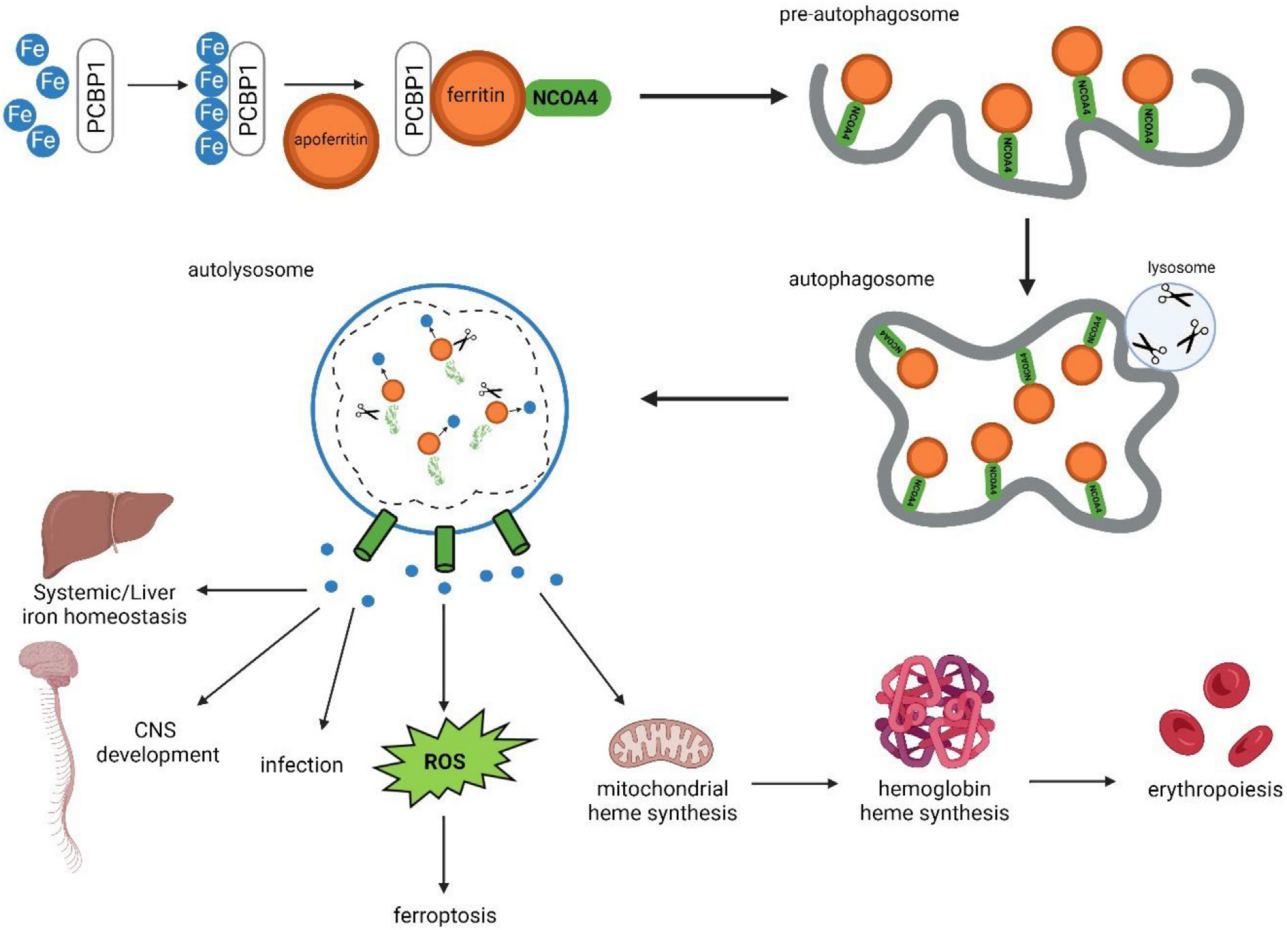
Ferritinophagy occurs to maintain basal iron homeostasis and during times of iron depletion. Ferritinophagy is initiated by the binding of nuclear receptor coactivator 4 (NCOA4) to ferritin, trafficking NCOA4 to the pre-autophagosome^130^. The mature autophagosome then fuses with a lysosome which mediates the degradation of ferritin to release iron^130^. In the cytosol, iron is utilized for physiologic processes such as liver iron homeostasis, central nervous system development, and erythropoiesis. However, increased ferritinophagy flux and iron overload can lead to reactive oxygen species (ROS) production and ferroptosis in addition to supporting proliferation of infectious bacteria^133^^,^^134^.

**Figure 6:**
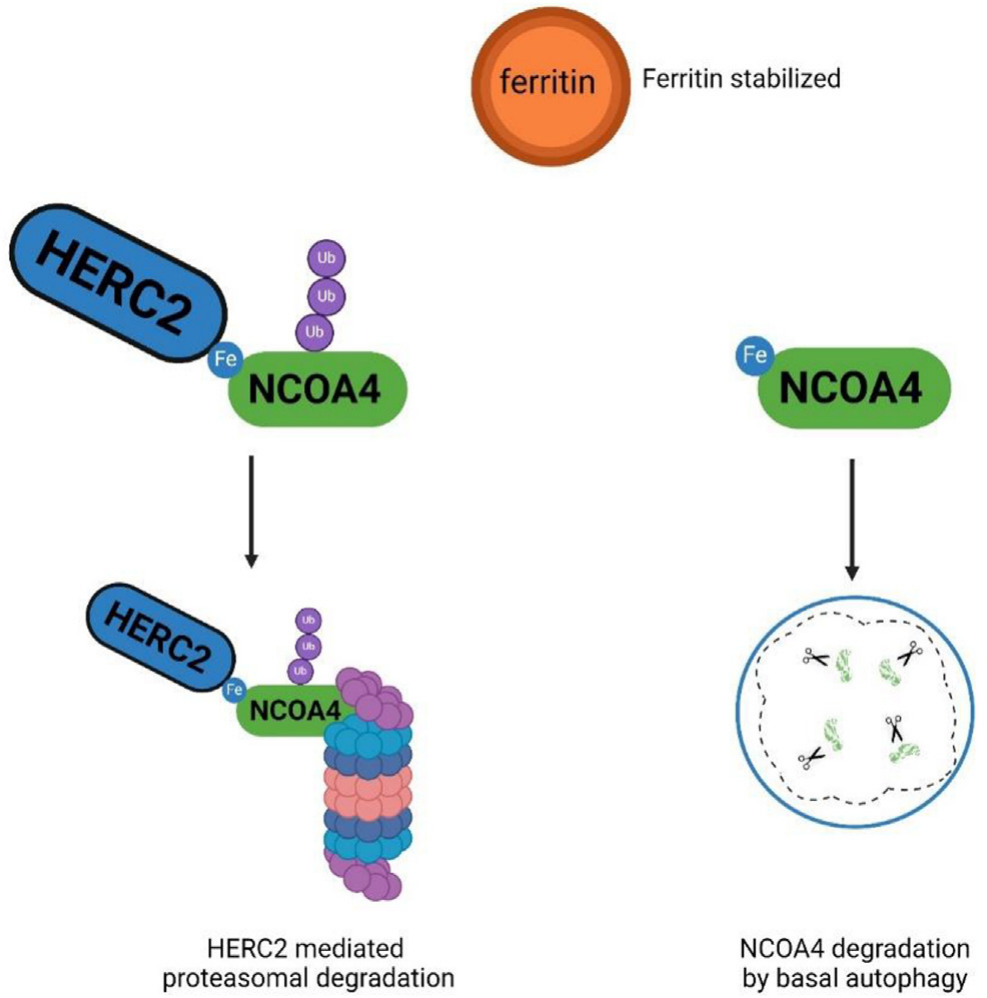
In an Iron-replete state, elevated intracellular iron levels result in iron-dependent binding of nuclear receptor coactivator 4 (NCOA4) to HERC2, leading to its proteasomal degradation^132^. NCOA4 is also subject to degradation through basal autophagy^3^. Reduced NCOA4 levels contribute to ferritin stabilization, decreased ferritinophagy, and a subsequent decline in intracellular iron levels^132^.

Autophagy is an evolutionarily conserved catabolic cellular process that maintains basal intracellular homeostasis through the turnover of proteins and organelles (Fig. 5). This process is important for cellular growth and development in addition to cellular adaptation to stress^123^. Various studies have found that deletion of autophagic proteins led to intracellular accumulation of ubiquitinated proteins, suggesting the essential role of autophagy in protein turnover^124^. After iron is sequestered within ferritin (Figure 2, Figure 4, Figure 5), its utilization requires the proteolytic degradation of the ferritin (ferritinophagy) by lysosomes in a process dependent on autophagy^6^^,^^125^^,^^126^. The intracellular concentration of iron is tightly regulated and mediated by ferritin, a ubiquitous protein complex composed of 24 subunits of ferritin light chains and FTH1^127^, capable of chelating up to 4500 atoms of Fe^3+^ iron^128^. Poly-rC binding protein (PCBP1), an iron chaperone, binds and delivers Fe^2+^ iron to a ferritin pore where it enters and is oxidized to the Fe^3+^ state by the iron oxidase activity of FTH1, thereby sequestering and storing iron in an inert form where it cannot participate in cellular activity and form harmful ROS^5^^,^^129^. Cargo-selective receptor proteins target specific organelles and pathogens for autophagic degradation. The cargo receptor for ferritin was identified in 2014 by Mancias et al.^130^ Their study identified nuclear receptor coactivator 4 (NCOA4), elucidating its role as the key mediator of ferritinophagy by binding ferritin at its C-terminal domain and delivering it to the pre-autophagosome (Fig. 5). More studies have also described an alternative pathway for lysosomal delivery of NCOA4-ferritin complexes *via* the endosomal pathway/endosomal sorting complex required for transport^131^. Mancias and co-authors also identified the role of HERC2 and NEURL4 proteins, subunits of an E3 ubiquitin ligase complex, which bind NCOA4 and lead to its subsequent proteasomal degradation^130^. During times of iron starvation or maintenance of basal iron levels NCOA4 binding to HERC2 decreases, leading to NCOA4 stabilization, induction of ferritinophagy, and iron release^3^^,^^132^. In times of iron excess NCOA4 binding to HERC2 (Fig. 6) increases leading to the proteasomal degradation of NCOA4, inhibiting ferritinophagy, and increasing ferritin iron storage^132^. In the cytosol, released iron is utilized for physiologic processes such as liver iron homeostasis and central nervous system development. However, increased ferritinophagy flux is also associated with ROS production leading to ferroptosis and has been shown to support the proliferation of uropathogenic *Escherichia coli* in urinary tract infections and predisposes severe infection with siderophilic bacteria^133^^,^^134^. At present time, ferritinophagy does not appear to participate in the pathogenesis of CRC. A study by Hasan et al. hypothesized ferritinophagy is essential for CRC proliferation due to high levels of iron required for cancer cell growth and oncogenic signaling^135^. This hypothesis was tested utilizing a CRISPR/Cas9 *NCOA4* knockout model in HCT116 and SW480 colon cancer cell lines compared to a wild-type NCOA4 model and examined growth under iron-deficient and iron-replete conditions, and the authors concluded that ferritinophagy is not required for basal cellular growth in CRC-derived cells^135^. Given the association of increased iron levels with intestinal inflammation, attenuating ferritinophagy would appear to be a therapeutic strategy for treating IBD, but to date, no study has been conducted on this subject.

### 6.3 Iron transportation

Since iron is required for the generation of lipid peroxides and initiating ferroptosis, compounds that affect intracellular iron levels can impact ferroptosis sensitivity^15^. NRAS-mutant fibrosarcoma cells co-treated with the ferroptosis inducer Erastin and DFO showed suppressed ROS accumulation and cell death by reducing intracellular LIP, thereby decreasing ferroptosis sensitivity^15^. Furthermore, cells with oncogenic RAS mutations exhibit increased iron content compared to normal cells. This is attributed to the upregulation of the transferrin receptor (increases iron import, Figure 2, Figure 4, Figure 5) and the downregulation of FTH1 and FTL (decreased iron storage). This increased iron content indicates heightened sensitivity to ferroptosis^13^. Given that NCOA4 directly modulates ferritin levels and intracellular iron levels, NCOA4-mediated ferritinophagy has also been demonstrated to influence ferroptosis sensitivity^136^^,^^137^. Knockdown of *NCOA4* in human pancreatic cancer and fibrosarcoma cell lines led to reduced levels of Fe^2+^ and malondialdehyde (MDA), a marker of oxidative stress and a product of lipid peroxidation, along with a simultaneous increase in GSH. Conversely, overexpression of *NCOA4* resulted in elevated levels of Fe^2+^ and MDA, along with decreased GSH^136^. Additionally, hepatic stellate cells from fibrotic patients, when treated with the ferroptosis inducer sorafenib (Table 3), exhibited an upregulation of *NCOA4* expression and a significant decrease in the expression of SQSTM1 and FTH1, both of which are substrates for ferritinophagy^138^. In summary, multiple sources consistently support the role of NCOA4 as a modulator of ferroptosis by promoting ferritinophagy-mediated iron release and ROS accumulation. These findings underscore the significance of iron and GSH in influencing metabolic redox activity, thereby either inducing or inhibiting ferroptosis.

### 6.4 Long non-coding RNA (lncRNA)

LncRNA research provides a multifaceted approach to cancer with potential applications for diagnosis, prognosis, and therapy. Since its discovery in the early 1990s, lncRNA was found to play a key role in gene regulation and expression in addition to influencing longevity pathways by regulating cellular proliferation, differentiation, and apoptosis^139^. LncRNAs are unique in function and purpose. They are post-transcriptionally modified, non-coding RNA strands greater than 200 nucleotides in length and are capable of forming RNA–protein complexes that regulate epigenetic processes^140^. LncRNAs are also differentially expressed across tissues, promote tumor suppressing or oncogenic activity, and have recently been shown to induce ferroptosis^141^. A 2018 study by Mao et al.^142^ uncovered a previously unknown function of a specific lncRNA P53RRA that was downregulated in cancers and shown to have a tumor suppressing effect by increasing retention of p53 in the nucleus resulting in increased lipid ROS and iron concentrations, thereby triggering ferroptosis. In similar fashion, a 2019 study by Qi et al.^143^ found that erastin upregulated lncRNA GABPB1-AS1, which downregulated protein GABPB1 resulting in downregulation of Peroxiredoxin-5 and sensitized hepatocellular carcinoma cells to ferroptosis. Qi's results demonstrated the prognostic value of lncRNAs by showing improved survival of HCC patients was correlated with high levels GABPB1-AS1 while simultaneously showcasing its use as a therapeutic target *via* erastin. With an estimated 16,000 to 100,000 lncRNAs within the human genome, many of which have an unknown function^144^, uncovering additional lncRNAs and their functions remains a growing topic of research for those studying ferroptosis' role in cancer.

## 7 Concluding remarks

Ferroptosis is a novel type of regulated cell death driven by intracellular accumulation of iron catalyzing formation of ROS with an insufficient capacity of thiol-dependent or antioxidant mechanisms to eliminate organic hydroperoxides. In this view, the dual role of iron is explored. As an essential micronutrient, iron serves as a cofactor for various metabolic processes. However, iron's electrochemical properties also cause its deleterious effects of catalyzing redox cycling reactions and free radical formation in addition to contributing to tumor initiation and growth. To maintain iron homeostasis, the protein ferritin provides a storage mechanism to sequester intracellular iron in an inert form. When iron levels are low, an autophagic-lysosomal process degrades ferritin to release iron in a process known as ferritinophagy, which is a modulator of ferroptosis sensitivity by controlling the degradation of ferritin *via* the action of cargo receptor NCOA4. In light of iron dysregulation's role in progression of disease and in ferroptosis, new research has implicated ferroptotic mechanisms in various disorders, including intestinal disease. Herein, the growing prevalence and associated mortality and morbidity of IBD and CRC are discussed, compelling exploration of novel approaches for treatment. The induction of ferroptosis has been found to halt the progression and eliminate malignant cells in various cancers, including CRC. Contrarily, the inhibition of ferroptosis for the treatment of IBD is essential to prevent inflammation and destruction of IECs and preserve the intestinal mucosal barrier.

Future investigations in modulating ferroptosis as a therapeutic approach for disease will focus on finding molecules with greater stability *in vivo* as addressed by Zhang et al.^54^ and propose more accurate assays to assess the degree of ferroptotic cell death, particularly for cancer treatment. This will be crucial as GPX4 seems to be a key component in ferroptosis but its function seems ambivalent depending on the cancer line it is studied in. In contrast, there do seem be a variety of promising modulators, reviewed here, that seem to have their roles resolved in ferroptosis. Furthermore, there are currently drugs such as the previously described RSL3, RSL5, and cisplatin that already modulate ferroptosis. These drugs as well as the vitamin K derivatives could serve as potential platforms for the development of therapeutic drugs with greater efficacy and selectivity. Finally, as genomic work advances prospects like the lncRNA may provide key insights in ferroptosis pathway that may provide more therapeutic avenues to explore.

In summary, while the majority of this article has focused on drug therapies and developing technologies in relation to ferroptosis and gastrointestinal disease, a mention of preventive health is warranted considering the changing epidemiology of IBD and CRC over time, geography, and environmental factors such as microplastics particularly in developing countries are heavily implicated in the rising incidence of disease^145^. When considering gastrointestinal disease from the lens of preventive health, the role of ferroptosis is obfuscated when absence of disease is not easily attributable to a specific molecular mechanism. However, the normal physiological role of ferroptosis remains poorly understood. At present moment, there is a paucity of research in preventive health of IBD and CRC with regards to ferroptosis. Future studies within this scope may further elucidate the physiologic role of ferroptosis and promote greater awareness surrounding gastrointestinal disease prevention and treatment.

## Declaration of Generative AI and AI-assisted technologies in the writing process

During the preparation of this work the authors used ChatGPT3.5 to improve language and readability. After using this tool/service, the authors reviewed and edited the content as needed and take full responsibility for the content of the publication.

## Acknowledgments

Christian V Cabanlong was financially supported by the NIGMS funded Academic Science Education and Research Training (ASERT, K12-GM088021) Program at the University of New Mexico Health Sciences Center. Xiang Xue received partial funding support from the National Institutes of Health (P20 GM130422), a Research Scholar Grant from the American Cancer Society (RSG-18-050-01-NEC), Environmental Health and Toxicology Pilot Awards from UNM Center for Native Environmental Health Equity Research (P50 MD015706), and New Mexico Integrative Science Program Incorporating Research in Environmental Sciences (NM-INSPIRES, 1P30ES032755). Xiang Xue also acknowledges funding support from a Research Program Support Pilot Project Award from UNM comprehensive cancer center (P30CA118100) and the Cardiovascular and Metabolic Disease Research Program Pilot Project Grant from UNMHSC Office of Research Signature Programs.

## Author contributions

Aaron T. Kao: Writing – original draft, Conceptualization. Christian V. Cabanlong: Writing – review & editing, Conceptualization. Kendra Padilla: Writing – review & editing. Xiang Xue: Writing – review & editing, Supervision, Project administration, Funding acquisition, Conceptualization.

## Conflicts of interest

The authors declare no conflicts of interest.
